# Exploring the impact of pupil expansion techniques on cataract surgery: unveiling key complications and clinical outcomes: a comparative analysis of 1266 eyes

**DOI:** 10.1007/s00417-025-06748-2

**Published:** 2025-02-03

**Authors:** Asaf Achiron, Tal Yahalomi, Michael Ostrovsky, Eliya Levinger, Eyal Cohen, Omar Elhaddad, Derek Tole, Kieren Darcy, Raimo Tuuminen

**Affiliations:** 1 Tel Aviv Sourasky Medical Center, Tel Aviv, Israel and Sackler School of Medicine, Tel Aviv University, Tel Aviv, Israel; 2 Department of Ophthalmology, Samson Assuta Ashdod Hospital and the faculty of Health Sciences, Ben-Gurion University of the Negev, Ashdod, Israel; 3 Bristol Eye Hospital, University Hospitals Bristol NHS Foundation Trust, Bristol, United Kingdom; 4 Faculty of Medicine, Alexandria University, Alexandria, Egypt; 5 Helsinki Retina Research Group, Faculty of Medicine, University of Helsinki, Helsinki, Finland; 6 Department of Ophthalmology, Kymenlaakso Central Hospital, Kotka, Finland

**Keywords:** Cataract surgery, Posterior capsular rupture, Pseudophakic cystoid macular edema, Pupil expansion, Zonular dialysis

## Abstract

**Background:**

In cataract surgery, inadequate pupil dilation presents a major surgical challenge by narrowing the operation field and restricting visibility and movement. We aim to compare cataract surgery complication rates and clinical outcomes using different pupil expansion methods.

**Methods:**

This retrospective cohort study grouped patients according to four techniques of mechanical pupil expansion techniques: sphincterotomy (*N* = 339), iris stretching (*N* = 242), iris hooks (*N* = 391) and expansion rings (*N* = 294). Incidences and odds ratios for major complications and outcomes were compared between the groups.

**Results:**

This single-center study included 1266 adult patients who underwent routine cataract surgery with mechanical pupil dilatation. The mean (± SD) age was 75.5 (± 13.0) years and 727 (57%) patients were male. The risk of pseudophakic cystoid macular edema (PCME) did not differ between the groups. Iris hooks were associated with the highest incidence of posterior capsular rupture (PCR) (3.3%) as compared to sphincterotomy, stretching and expansion rings (0.9%, 0.4% and 1.4%, respectively, *P* = 0.016). However, this effect was not supported by multivariable analysis. Zonular dialysis tended to be higher among eyes operated with iris hooks and pupil expansion rings, compared with iris stretching and sphincterotomy (2.0% and 1.7%, respectively, *P* = 0.058) and was found to be independently associated with a specific mechanical pupil expansion method on multivariable analysis (*P* = 0.050). No differences were observed for other complications, intraocular pressure or best-corrected visual acuity (VA) gain. Surgeon seniority was a significant protective factor from postoperative uveitis on multivariable analysis (*P* = 0.032).

**Conclusions:**

Our large cohort study found no difference between the groups regarding major complications or clinical outcomes, suggesting that all four methods may be equally safe.

**Key messages:**

*****What is known***:**

• In cataract surgery, inadequate pupil dilation presents a major surgical challenge by narrowing the operation field and restricting visibility and movement.

• Different pupil dilation methods have been used, ranged from topical and intracameral mydriatics and visco-mydriasis to mechanical dilation maneuvers.

• Four principal techniques of mechanical pupil expansion, including sphincterotomies, manual iris stretching, iris retracting hooks and pupil expansion rings, are available.

*****What is new***:**

• This single-center study included 1266 adult patients found no difference between the groups regarding major complications or clinical outcomes such as pseudophakic cystoid macular edema (PCME), posterior capsular rupture, zonular dialysis, intraocular pressure, uveitis or best-corrected visual acuity gain.

• Surgeon seniority was a significant protective factor from postoperative uveitis.

**Supplementary Information:**

The online version contains supplementary material available at 10.1007/s00417-025-06748-2.

## Introduction

According to the WHO, nearly 100 million people worldwide suffer from moderate to severe vision impairment due to cataracts, with approximately 20 million surgeries performed annually [1].As cataract surgery is considered one of the most successful interventions in contemporary medicine, patients' expectations of consistently successful, complication-free procedures for cataracts are rising.

In cataract surgery, inadequate pupil dilation presents a major surgical challenge by narrowing the operation field and restricting visibility and movement. Large case series have previously found between 4–7% of surgeries to be associated with a small pupil, often secondary to local or systemic conditions such as synechia, uveitis, previous surgery or trauma, diabetes, intraoperative floppy iris syndrome (IFIS), pseudoexfoliation syndrome (PXF) or chronic mitotic therapy [2–5]. Miosis may also occur intraoperatively, for instance, due to prostaglandin release during femtosecond laser-assisted cataract surgery (FLACS) [6]. Small pupil has been linked to increased complication rates, including anterior or posterior capsule rupture, retained lens material, unconventional intraocular lens (IOL) placement, dropped nucleus, iris trauma, hyphema, vitreous loss, pseudophakic cystoid macular edema (PCME), postoperative inflammation, retinal detachment, and damage to the corneal epithelium [4, 7, 8].

**Fig. 1:**
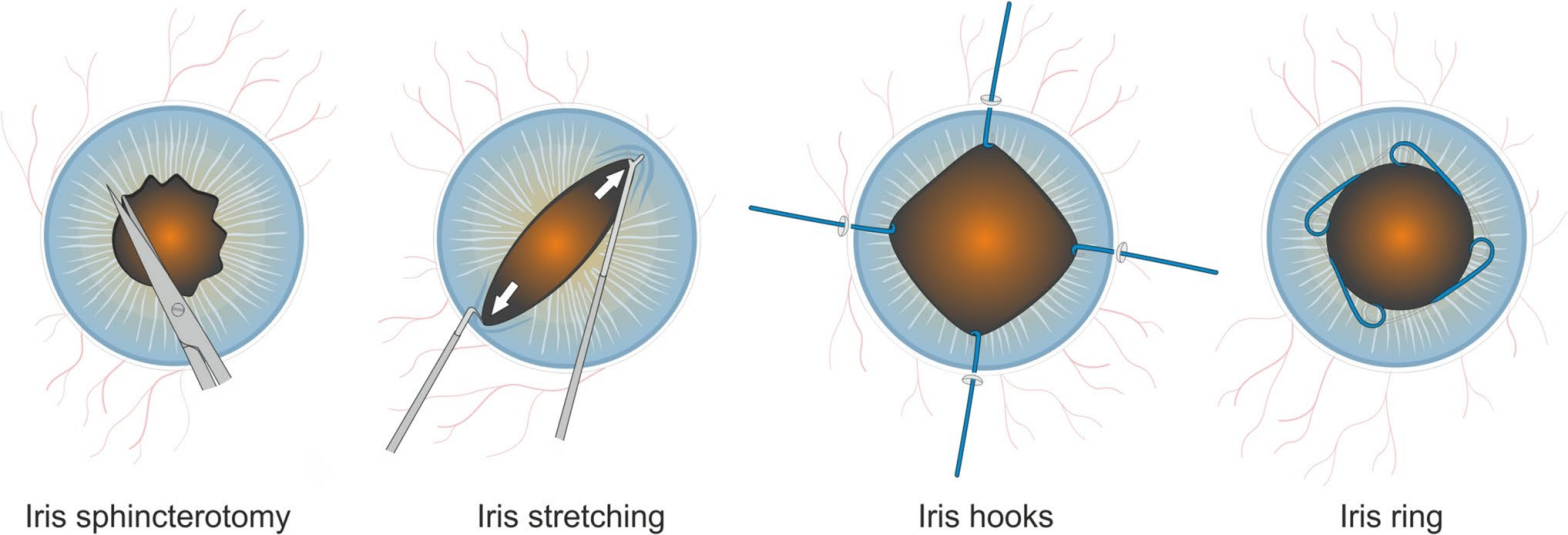
Illustration of different mechanical pupil expansion methods

Different pupil dilation methods have been used to address small pupil cases. These range from topical and intracameral mydriatics and visco-mydriasis to cases where pharmacological strategies fail and mechanical dilation maneuvers are necessary [2, 4]. Four principal techniques of mechanical pupil expansion, including sphincterotomies, manual iris stretching, iris retracting hooks and pupil expansion rings, are available [5] (Fig. 1). While these techniques are generally safe and effective in minimizing complications stemming from small pupils, different methods may vary regarding added surgical time and intra- or postoperative complications, thus affecting clinical outcomes [3, 9].

Overall, there is a lack of comparative data regarding mechanical pupil dilation techniques, as shown in Supplemental Table 1. Thus, our study aimed to compare complications and clinical outcomes between four mechanical pupil expansion methods: sphincterotomies, manual stretching, iris retracting hooks and pupil expansion rings.

**Table 1:** Baseline parameters according to pupil expansion method

	**Sphincterotomy** *N* = 339	**Stretching** *N* = 242	**Hooks** *N* = 391	**Expansion ring** *N* = 294	*P*-value
Age (years)	78.2 ± 11.8	77.1 ± 10.7	71.5 ± 16.0	76.2 ± 12.2	** < 0.001**
Sex (M:F)	187:152	131:111	238:153	171:123	0.286
Laterality (R:L)	161:178	107:135	209:182	145:149	0.131
IOP (mmHg)	16.3 ± 5.9	15.6 ± 4.6	16.0 ± 5.2	16.0 ± 4.8	0.566
BCVA (LogMAR)	0.82 ± 0.60	0.70 ± 0.61	0.87 ± 0.68	0.77 ± 0.63	**0.002**
Axial length (mm)	23.2 ± 1.3	23.3 ± 1.2	23.2 ± 1.7	23.1 ± 1.4	0.511
NSAID post-op	6 (1.8%)	2 (0.8%)	12 (3.1%)	17 (5.8%)	**0.003**
**Co-morbidity**					
AMD	23 (6.8%)	10 (4.1%)	16 (4.1%)	11 (3.7%)	0.228
DM (type 1:2)	6:75 (1.8%:22.7%)	8:68 (3.6%:30.2%)	13:74 (3.7%:21.2%)	8:85 (2.7%:28.9%)	**0.009**
DR	21 (6.2%)	17 (7.0%)	30 (7.7%)	25 (8.5%)	0.714
ERM	14 (4.1%)	13 (5.4%)	21 (5.4%)	15 (5.1%)	0.865
Glaucoma	93 (27.4%)	58 (24.0%)	102 (26.1%)	89 (30.3%)	0.389
PGA	71 (20.9%)	48 (19.8%)	74 (18.9%)	67 (22.8%)	0.636
RVO	4 (1.2%)	1 (0.4%)	1 (0.3%)	2 (0.7%)	0.663
Uveitis	30 (8.8%)	22 (9.1%)	37 (9.5%)	24 (8.2%)	0.905
Vitrectomy	3 (0.9%)	4 (1.7%)	9 (2.3%)	3 (1.0%)	0.384
**Surgeon experience**					
Consultant	290 (85.5%)	216 (89.3%)	261 (66.8%)	169 (57.7%)	
Specialist	15 (4.4%)	9 (3.7%)	51 (13.0%)	20 (6.8%)	
Fellow	13 (3.8%)	10 (4.1%)	59 (15.1%)	54 (18.4%)	
Trainee	21 (6.2%)	7 (2.9%)	20 (5.1%)	51 (17.3%)	** < 0.001**

Data are given as mean (± SD) or absolute values (and proportions). Multiple groups were compared with the Fisher-Freeman-Halton test for qualitative data, with the Kruskall-Wallis for non-parametric variables and with the one-way ANOVA test using Bonferroni correction for continues variables. IOP; intraocular pressure, BCVA; best-corrected visual acuity, logMAR; logarithm of the minimum angle of resolution, NSAID; non-steroidal anti-inflammatory drug, AMD; age-related macular degeneration, DM; diabetes mellitus, DR; diabetic retinopathy, ERM; epiretinal membrane, PGA; prostaglandin analogue, RVO; retinal vein occlusion

## Methods

This was a registry-based retrospective cohort study of consecutive adult cataract surgeries performed at the Department of Ophthalmology, Bristol Eye Hospital, Bristol, UK. Patients were enrolled between January 2013 and March 2022 and were admitted according to national cataract management guidelines. This study received the local ethics committee approval (CORN/SE/2021–2022/02 and was presented to the local audit authority) and adhered to the tenets of the Declaration of Helsinki.

**Fig. 2:**
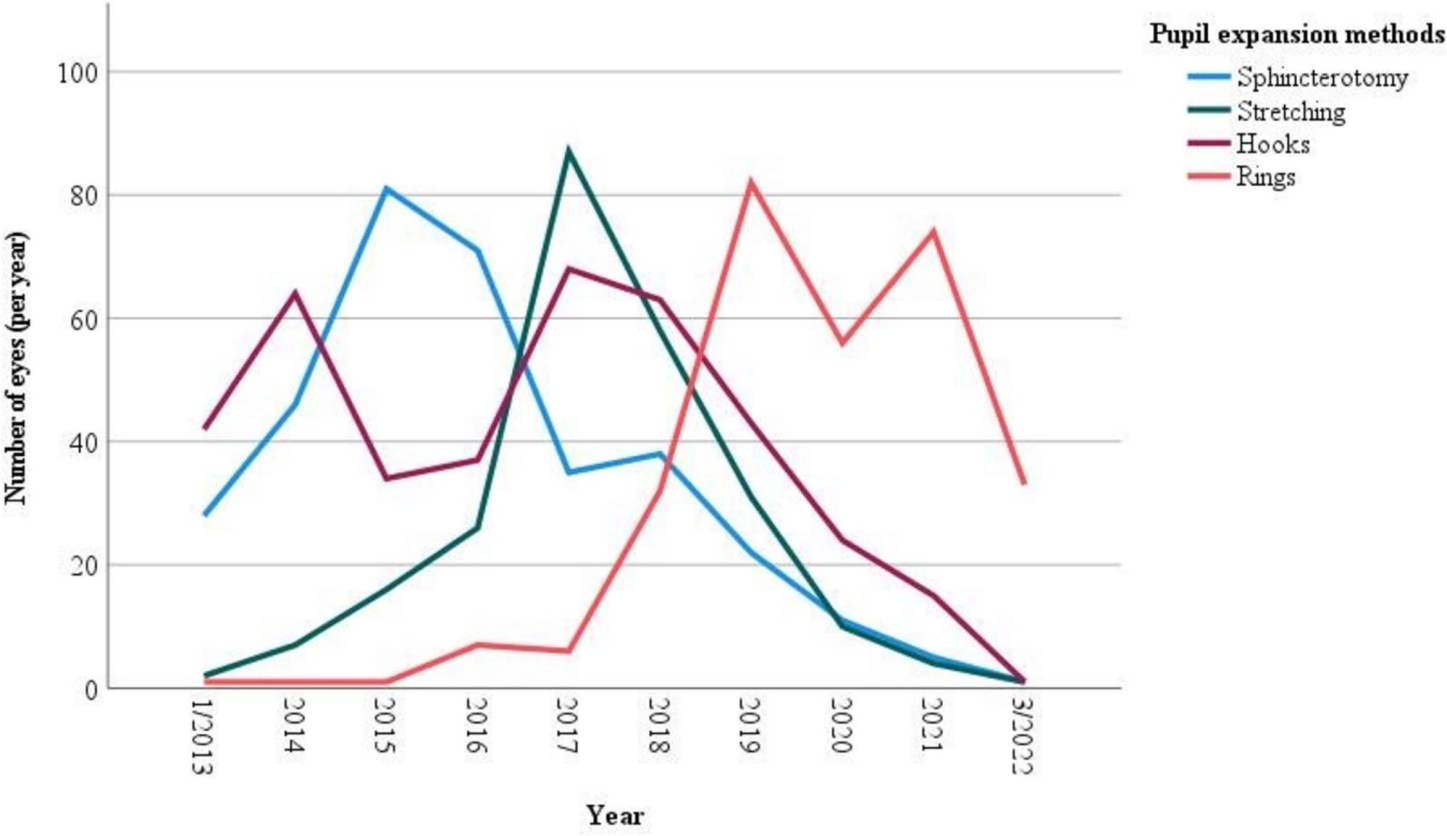
Number of operated eyes over the study period in regard to different mechanical pupil expansion methods

Inclusion criteria were all adult patients who underwent phacoemulsification surgery and intraocular lens implantation with mechanical pupil dilatation. Those methods included sphincterotomy (N = 339), stretching (N = 242), iris hooks (N = 391; MicroSurgical Technology/MST Inc., Redmond, WA) and pupil expansion rings (N = 294; Malyugin rings, MST Inc., Redmond, WA, and Morcher Pupil dilator Type 5S, FCI Ophthalmics, Pembroke, MA) (Fig. 2). All cases received standard topical and intracameral mydriatics.

### Data acquisition and subjects

Data was collected from the patient medical records system (Medisoft Ltd, Leeds, UK). Clinical variables were registered for age at surgery and sex (male/female), date of cataract surgery and laterality, DM status (no/type1/type2), the axial length of the eye, best-corrected visual acuity (BCVA, transformed into logMAR units) and intraocular pressure (mmHg) preoperatively and within 3 months postoperatively, ocular co-morbidities (age-related macular degeneration, diabetic retinopathy, epiretinal membrane, glaucoma and use of prostaglandin analogs, retinal vein occlusion, uveitis, previous vitrectomy), surgeon seniority (trainee/fellow/specialist/consultant), and the incidence of intra- and postoperative cataract surgery complications, such as uveitis and PCME. Post operative uveitis was defined as a persistent anterior chamber cells following a normal surgery course, or a much more severe anterior chamber cells appearance than expected, all despite standard post operative drops.

### Statistical analyses

Data are presented as mean ± standard deviation (SD) or absolute values and proportions. Statistical analysis was performed using IBM SPSS Statistics 27 (IBM SPSS Statistics for Windows, Version 27.0. IBM Corp., Armonk, NY). Multiple groups were compared with the Fisher-Freeman-Halton test for qualitative data, the Kruskall-Wallis test for non-parametric variables, and the one-way ANOVA test using Bonferroni correction for continuous variables. Odds ratios with 95% CIs for the intra- and postoperative complications were calculated using multivariable regression analysis, having age, sex, diabetes, and surgeon seniority as confounders. Postoperative topical non-steroidal anti-inflammatory drug (NSAID) use was ignored due to its significant collinearity with diabetes and small sample size. P-values ≤ 0.05 were considered statistically significant.

## Results

Included were 1266 eyes operated with mechanical pupil dilatation. The mean age of patients operated with iris hooks (71.5 ± 16.0 years) was lower than those operated with sphincterotomy (78.2 ± 11.8 years), stretching (77.1 ± 10.7 years) or pupil expansion ring (76.2 ± 12.2 years) (P < 0.001, Table 1). Baseline BCVA in the operated eyes (P = 0.002), the use of NSAIDs postoperatively (P = 0.003) and the prevalence of diabetes type 1 and 2 among patients (P = 0.009) differed between the study groups. Furthermore, proportional surgeon seniority differed significantly between the pupil expansion methods (P < 0.001).

### Complications and clinical outcomes

The use of iris hooks was significantly associated with the highest incidence of posterior capsular rupture (3.3% compared with 0.4–1.4% for other methods, P = 0.016, Table 2). Zonular dialysis tended to be higher among eyes operated with iris hooks (2.0%) and pupil expansion ring (1.7%), compared with sphincterotomy (0%) and iris stretching (0.8%) (P = 0.058, Table 3). No differences were observed for choroidal hemorrhage, corneal edema, dropped nucleus, hyphema and iris prolapse.

**Table 2:** Intra-operative complications and clinical outcomes according to pupil expansion method

	**Sphincterotomy** *N* = 339	**Stretching** *N* = 242	**Hooks** *N* = 391	**Expansion ring** *N* = 294	*P*-value
**Complication**					
PCR	3 (0.9%)	1 (0.4%)	13 (3.3%)	4 (1.4%)	**0.016**
Zonular dialysis	0	2 (0.8%)	8 (2.0%)	5 (1.7%)	0.058
Choroidal hemorrhage	0	1 (0.4%)	0	1 (0.3%)	0.427
Corneal edema	0	1 (0.4%)	2 (0.5%)	3 (1.0%)	0.318
Dropped nucleus	2 (0.6%)	0	3 (0.8%)	0	0.281
Hyphema	0	1 (0.4%)	2 (0.5%)	1 (0.3%)	0.653
Iris prolapse	5 (1.5%)	4 (1.7%)	11 (2.8%)	3 (1.0%)	0.325
**Clinical outcomes**					
PCME	7 (2.1%)	3 (1.2%)	12 (3.1%)	6 (2.0%)	0.484
IOP (mmHg)	14.7 ± 5.6	14.4 ± 5.2	14.7 ± 5.6	14.8 ± 4.9	0.963
BCVA (logMAR)	0.40 ± 0.53	0.25 ± 0.37	0.45 ± 0.58	0.33 ± 0.48	**0.001**
BCVA gain (logMAR)	0.31 ± 0.50	0.40 ± 0.53	0.35 ± 0.48	0.34 ± 0.50	0.405
Uveitis	15 (4.4%)	14 (5.8%)	27 (6.9%)	18 (6.1%)	0.554
Anti-VEGF	13 (3.8%)	12 (5.0%)	20 (5.1%)	11 (3.7%)	0.755
Intravitreal steroid	8 (2.4%)	2 (0.8%)	8 (2.0%)	1 (0.3%)	0.117

Data are given as mean (± SD) or absolute values (and proportions). Multiple groups were compared with the Fisher-Freeman-Halton test for qualitative data, with the Kruskall-Wallis for non-parametric variables and with the one-way ANOVA test using Bonferroni correction for continues variables. PCR; posterior capsule rupture, IOP; intraocular pressure, BCVA; best-corrected visual acuity, logMAR; logarithm of the minimum angle of resolution, VEGF; vascular endothelial growth factor, PCME; pseudophakic cystoid macular edema

**Table 3:** multivariable analysis of odds ratios with 95% CIs for cataract surgery outcomes

**Factor**	Age (y)	Sex (M:F)	Diabetes (yes:no)	Surgeon seniority	Pupil expansion method
Posterior capsule rupture	1.017 (0.985–1.050) P = 0.313	0.391 (0.126–1.217) P = 0.105	1.876 (0.865–4.066) P = 0.111	0.706 (0.463–1.079) P = 0.108	0.748 (0.464–1.204) P = 0.231
Zonular dialysis	1.002 (0.94–1.042) P = 0.919	3.677 (1.138–11.88) P = **0.030**	0.955 (0.524–1.740) P = 0.879	0.860 (0.534–1.386) P = 0.536	0.573 (0.328–1.000) P = **0.050**
Uveitis	1.069 1.053–1.085 P < **0.001**	1.556 (0.909–2.661) P = 0.107	1.120 (0.805–1.558) P = 0.502	0.755 (0.583–0.976) P = **0.032**	1.078 (0.838–1.386) P = 0.559
PCME	1.002 0.973–1.031 P = 0.912	0.837 0.375–1.867 P = 0.664	1.203 0.739–1.958 P = 0.458	1.122 0.722–1.745 P = 0.608	0.876 0.610–1.259 P = 0.475

PCME; pseudophakic cystoid macular edema

Rates of PCME did not significantly differ between the groups, ranging between 1.2% in the stretching group, 2.0% and 2.1% in the expansion ring and sphincterotomy groups, respectively, and 3.1% in the iris hooks group (*P* = 0.484, Table 3). Postoperative BCVA was best among eyes with iris stretching (0.25 ± 0.37 logMAR units), compared to eyes with sphincterotomy, iris hooks and pupil expansion rings (0.40 ± 0.53, 0.45 ± 0.58, and 0.33 ± 0.48 logMAR units, respectively, *P* = 0.001, Table 3). BCVA gain, however, non-significantly ranged from 0.31 to 0.40 logMAR units between the different mechanical pupil expansion methods (*P* = 0.405). No differences between the groups were observed for postoperative uveitis or for IOP (*P* = NS, Table 3).

In multivariable regression analysis, PCME, PCR and uveitis were not found to be independently associated with the method of mechanical pupil expansion (*P* = 0.475, *P* = 0.231 and *P* = 0.559, respectively, Table 3). However, zonular dialysis was found to be independently associated with the method of mechanical expansion (*P* = 0.050).

## Discussion

In this study, we assessed the risk for major intra and postoperative complications and the clinical outcomes of a large cohort of adult cataract patients, operated with pupil expansion by one of four methods: sphincterotomy, stretching, iris hooks or pupil expansion rings. To the best of our knowledge, this is the first study to compare all four principal techniques for mechanical pupil expansion. Despite several other differences between the groups, our data do not support an association between the method of pupil expansion and PCME. Notably, zonular dialysis was more commonly associated with the use of iris hooks and pupil expansion rings.

During the last decade, pupil expansion rings gradually became the predominant mechanical expansion method by residents and specialists alike, as seen in our data and several other recent studies [2, 3]. While small pupils have been associated with several intraoperative complications, such as iris trauma and bleeding or postoperative inflammation, it should be noted that mechanical techniques to manage a small pupil are also not complication-free [5, 7, 10–13]. Previous reports have noted several key differences between mechanical expansion techniques. For instance, Akman et al. showed iris retracting hooks and polymethyl methacrylate pupil dilator rings to be more time-consuming but more stable throughout surgery when compared to manual stretching [9]. Regarding intraoperative complications, for example, it has been suggested that manual stretching may lead to micro-tears in the iris sphincter, potentially resulting in permanent mydriasis, as well as hyphema in susceptible eyes. Therefore, sphincterotomies may be indicated in very small or fibrotic irides [14]. Wang et al. found corneal endothelial cell density (ECD) to be higher after pupil expansion using iris hooks and Malyugin ring than bimanual stretching and radial cutting [15]. In contrast, Wilczynski et al. found more ECD loss using iris hooks than the Malyugin ring [16].

A study by Wilczynski et al. found that pupil expansion with Malyugin ring manifested in better postoperative BCVA than expansion using iris hooks [16]. Similarly, BCVA in our study was best among eyes operated with iris stretching (P = 0.001), followed by pupil expansion rings, sphincterotomy and iris hooks. However, it is important to note that these results follow the same trend as the baseline BCVA in our study, which differed significantly and was also best in the iris stretching group. BCVA gain ranged between 0.42 and 0.45 logMAR units with no significant differences between the pupil expansion methods. A large study by Balal et al. recently compared iris hooks, Malyugin ring and intracameral phenylephrine as management options in small pupil cases. It found no difference in VA improvement between the different methods when independent variables were controlled for [2].

A study by Nderitu and Ursell found that the use of Malyugin ring resulted in postoperative anterior uveitis and corneal edema, compared to iris hooks or no pupil expansion [3]. Substantial pupil expansion with iris hooks has also been associated with PCME and chronic inflammation, among other postoperative complications [17]. In our study, however, all postoperative complications were observed to be similar between the groups. This was further substantiated in multivariable regression analysis, as neither PCME nor postoperative uveitis were independently associated with any specific mechanical pupil expansion method. There was a higher proportion of consultant-level surgeons in the sphincterotomy and stretching groups than in the iris hooks and expansion ring groups. Fellows used iris hooks and expansion rings more commonly, while trainees used expansion rings more than any other method.

Regarding the confounders assessed in our study, multivariable analysis showed patient age to be an independent risk factor for postoperative uveitis, while surgeon seniority was a significant protective factor against it. In addition, male sex was found to be a risk factor for zonular dialysis. Although it was previously suggested that some of the pupil expansion methods (namely iris hooks and pupil expansion rings) might also assist the stabilization of the capsular bag, our results do not emphasize protection from zonular dialysis [14]. In fact, zonular dialysis in our study tended to be higher among eyes operated with iris hooks and pupil expansion rings. Of note, iris hooks were also associated with the highest incidence of posterior capsular rupture, which was insignificant in multivariable analysis. In addition, the iris hooks and expansion ring groups had a lower proportion of consultant-level surgeons. Still, there were also significant differences in patient age and diabetes as systemic comorbidity between the groups, which could have accounted for the difference.

This study includes a few limitations that must be considered. The study had a retrospective, non-randomized design and several differences were found between the groups’ baseline characteristics, which may confound the results. Moreover, selection bias should be considered, as both preoperative risk stratification and surgeon preference, which may affect the selection of the expansion method and lead to risk for complications independent from the expansion method selected, were not taken into account in the study. To mitigate this concern, multivariable analysis controlling for age, sex, diabetes mellitus and surgeon seniority was performed to assess the independent effect of specific pupil expansion methods on cataract surgery complications. Finally, the study is based on data from a single center with a homogenous population, which may lower the results’ external validity.

In conclusion, our analysis of 1266 eyes operated with different mechanical pupil expansion methods found no difference in the risk for PCME or PCR between the groups, and suggests that all techniques may be equally considered. Optimal pupil expansion method selection may be based on local clinical practices, surgeon preference, additional operative time required and available resources.

## Supplementary Information

Below is the link to the electronic supplementary material.

Supplementary file1 (DOCX 20 KB)

## Declarations

All procedures performed in studies involving human participants were in accordance with the ethical standards and with the 1964 Helsinki declaration and its later amendments or comparable ethical standards. This study received the local ethics committee approval (CORN/SE/2021–2022/02 and was presented to the local audit authority) and adhered to the tenets of the Declaration of Helsinki.

## References

[1] Steinmetz JD, Bourne RRA, Briant PS et al (2021) Causes of blindness and vision impairment in 2020 and trends over 30 years, and prevalence of avoidable blindness in relation to VISION 2020: the Right to Sight: an analysis for the Global Burden of Disease Study. Lancet Glob Health 9(2):e144–e160. 10.1016/S2214-109X(20)30489-7 33275949 10.1016/S2214-109X(20)30489-7 PMC7820391

[2] Balal S, Jbari AS, Nitiahpapand R et al (2021) Management and outcomes of the small pupil in cataract surgery: iris hooks, Malyugin ring or phenylephrine? Eye 35(10):2714–2718. 10.1038/s41433-020-01277-0 33184489 10.1038/s41433-020-01277-0 PMC8452752

[3] Nderitu P, Ursell P (2019) Iris hooks versus a pupil expansion ring: Operating times, complications, and visual acuity outcomes in small pupil cases. J Cataract Refract Surg 45(2):167–173. 10.1016/j.jcrs.2018.08.038 30527439 10.1016/j.jcrs.2018.08.038

[4] Malyugin BE (2018) Recent advances in small pupil cataract surgery. Curr Opin Ophthalmol 29(1):40–47. 10.1097/ICU.0000000000000443 29059105 10.1097/ICU.0000000000000443

[5] Hashemi H, Seyedian MA, Mohammadpour M (2015) Small pupil and cataract surgery. Curr Opin Ophthalmol 26(1):3–9. 10.1097/ICU.0000000000000116 25390859 10.1097/ICU.0000000000000116

[6] Abell RG, Darian-Smith E, Kan JB, Allen PL, Ewe SYP, Vote BJ (2015) Femtosecond laser–assisted cataract surgery versus standard phacoemulsification cataract surgery: Outcomes and safety in more than 4000 cases at a single center. J Cataract Refract Surg 41(1):47–52. 10.1016/j.jcrs.2014.06.025 25466483 10.1016/j.jcrs.2014.06.025

[7] Narendran N, Jaycock P, Johnston RL et al (2009) The Cataract National Dataset electronic multicentre audit of 55 567 operations: risk stratification for posterior capsule rupture and vitreous loss. Eye 23(1):31–37. 10.1038/sj.eye.6703049 18327164 10.1038/sj.eye.6703049

[8] Sparrow JM, Taylor H, Qureshi K, Smith R, Birnie K, Johnston RL (2012) The Cataract National Dataset electronic multi-centre audit of 55 567 operations: risk indicators for monocular visual acuity outcomes. Eye 26(6):821–826. 10.1038/eye.2012.51 22441022 10.1038/eye.2012.51 PMC3376295

[9] Akman A, Yilmaz G, Oto S, Akova YA (2004) Comparison of various pupil dilatation methods for phacoemulsification in eyes with a small pupil secondary to pseudoexfoliation. Ophthalmology 111(9):1693–1698. 10.1016/j.ophtha.2004.02.008 15350324 10.1016/j.ophtha.2004.02.008

[10] Sarnicola E, Sarnicola C, Sarnicola V (2023) Dilation devices in cataract surgery. Curr Opin Ophthalmol 34(1):71–77. 10.1097/ICU.0000000000000922 36484211 10.1097/ICU.0000000000000922

[11] Goh JWY, Harrison R, Tavassoli S, Tole DM (2018) Outcomes of sphincterotomy for small pupil phacoemulsification. Eye 32(8):1334–1337. 10.1038/s41433-018-0079-2 29618836 10.1038/s41433-018-0079-2 PMC6085369

[12] Taipale C, Holmström EJ, Ilveskoski L, Tuuminen R (2019) Incidence of pseudophakic cystoid macular edema in eyes with and without pupil expansion device. Acta Ophthalmol 97(7):688–694. 10.1111/aos.14007 30575287 10.1111/aos.14007

[13] Kreku R, Behndig A (2022) Consequences of mechanical pupil dilation, a study based on the Swedish national cataract register. Acta Ophthalmol 100(5):520–525. 10.1111/aos.15041 34596954 10.1111/aos.15041

[14] Grzybowski A, Kanclerz P (2020) Methods for achieving adequate pupil size in cataract surgery. Curr Opin Ophthalmol 31(1):33–42. 10.1097/ICU.0000000000000634 31743155 10.1097/ICU.0000000000000634

[15] da Wang J, Zhang JS, Li M, Mao YY, Mayinuer Y, Wan XH (2022) Comparison of different pupil dilatation methods for phacoemulsification in eyes with a small pupil. BMC Ophthalmol 22(1):173. 10.1186/s12886-022-02402-1 35436870 10.1186/s12886-022-02402-1 PMC9016963

[16] Wilczynski M, Wierzchowski T, Synder A, Omulecki W (2013) Results of Phacoemulsification with Malyugin Ring in Comparison with Manual Iris Stretching with Hooks in Eyes with Narrow Pupil. Eur J Ophthalmol 23(2):196–201. 10.5301/ejo.5000204 23112041 10.5301/ejo.5000204

[17] Masket S (1996) Avoiding complications associated with iris retractor use in small pupil cataract extraction. J Cataract Refract Surg 22(2):168–171. 10.1016/S0886-3350(96)80213-6 8656379 10.1016/s0886-3350(96)80213-6

