# Supplementary material for: Exploring the impact of pupil expansion techniques on cataract surgery: unveiling key complications and clinical outcomes: a comparative analysis of 1266 eyes
Source: Graefes Arch Clin Exp Ophthalmol. 2025 Feb 3;263(6):1597–603. doi: 10.1007/s00417-025-06748-2 (PMC12238154; doi:10.1007/s00417-025-06748-2)
Supplement: Supplementary file 1 — Supplementary file1 (DOCX 20 KB) [file 417_2025_6748_MOESM1_ESM.docx]

| Supplemental Table 1. Literature review of intra- and postoperative complications according to pupil expansion method. | | | | | | | | |
| --- | --- | --- | --- | --- | --- | --- | --- | --- |
|  | **Complication** | **Iris tear** | **PCR** | **Zonular  dialysis** | **Anterior uveitis** | **Corneal ECD loss** | **Corneal edema** | **PCME** |
| **Expansion method** |  |  |  |  |  |  |  |  |
| Sphincterotomy |  |  |  |  | 3.50% | 33.40% |  | 5.30% |
| Stretching |  |  |  |  |  | 41.70% |  |  |
| Iris hooks |  | 0.50% | 3.6%-4.2% | 1.10% | 1.1%-3.1% | 13.7-16.4% | 2.1%-15.5% | 1.1%-3.1% |
| Expansion ring |  | 1.30% | 1.6%-1.9% | 1.30% | 2.3%-6.7% | 9.4-25.2% | 7.0%-8.5% | 2.9%-3.2% |
| PCR; posterior capsule rupture, ECD; endothelial cell density, PCME; pseudophakic cystoid macular edema. | | | | | | | | |
